# Music expertise shapes audiovisual temporal integration windows for speech, sinewave speech, and music

**DOI:** 10.3389/fpsyg.2014.00868

**Published:** 2014-08-07

**Authors:** Hweeling Lee, Uta Noppeney

**Affiliations:** ^1^Cognitive Neuroimaging Group, Max Planck Institute for Biological CyberneticsTübingen, Germany; ^2^Memory Dysfunction in Neurodegenerative Diseases, German Center for Neurodegenerative Diseases (DZNE)Bonn, Germany; ^3^Computational Neuroscience and Cognitive Robotics Centre, School of Psychology, University of BirminghamBirmingham, UK

**Keywords:** multisensory, temporal synchrony, audiovisual integration, plasticity, speech, music

## Abstract

This psychophysics study used musicians as a model to investigate whether musical expertise shapes the temporal integration window for audiovisual speech, sinewave speech, or music. Musicians and non-musicians judged the audiovisual synchrony of speech, sinewave analogs of speech, and music stimuli at 13 audiovisual stimulus onset asynchronies (±360, ±300 ±240, ±180, ±120, ±60, and 0 ms). Further, we manipulated the duration of the stimuli by presenting sentences/melodies or syllables/tones. Critically, musicians relative to non-musicians exhibited significantly narrower temporal integration windows for both music and sinewave speech. Further, the temporal integration window for music decreased with the amount of music practice, but not with age of acquisition. In other words, the more musicians practiced piano in the past 3 years, the more sensitive they became to the temporal misalignment of visual and auditory signals. Collectively, our findings demonstrate that music practicing fine-tunes the audiovisual temporal integration window to various extents depending on the stimulus class. While the effect of piano practicing was most pronounced for music, it also generalized to other stimulus classes such as sinewave speech and to a marginally significant degree to natural speech.

## Introduction

Music training provides a rich multisensory experience that requires integrating signals from different sensory modalities with motor responses. Thus, the musician's brain provides an ideal model to study experience-dependent plasticity in humans (Munte, 2002; Zatorre et al., 2007). Previous research has shown that musicians develop an enhanced auditory system, both at the structural and functional levels (Schlaug et al., 1995; Munte et al., 2002; Schneider et al., 2005; Hannon and Trainor, 2007; Baumann et al., 2008; Imfeld et al., 2009) that seems to benefit linguistic and non-linguistic skills (Magne et al., 2006; Marques et al., 2007; Moreno et al., 2009; Tzounopoulos and Kraus, 2009; Kraus and Chandrasekaran, 2010). Specifically, musicians proved to be better than non-musicians at segmenting speech from background noise (Parbery-Clark et al., 2013), pitch (Besson et al., 2007), and prosodic tasks (Thompson et al., 2004).

Since practicing a musical instrument for an extensive period of time involves precise timing of several hierarchically organized actions, musical expertise may in particular influence the temporal binding of signals across the senses during perception. Even though sensory signals do not have to be precisely synchronous, they have to co-occur within a certain temporal integration window in order to be integrated into a unified percept (Stein et al., 1993; Spence and Squire, 2003; Noesselt et al., 2007, 2008; Lewis and Noppeney, 2010; Stevenson et al., 2011). Recent studies have shown that the temporal integration window can be narrowed or shifted via long-term musical training (Petrini et al., 2009), short-term perceptual learning (Powers et al., 2009), or short-term audiovisual exposure (Fujisaki et al., 2004). Conversely, it can be widened by exposure to asynchronous stimuli (Navarra et al., 2005).

One critical question is to which extent the impact of musical expertise on audiovisual synchrony perception is specific to the practiced music or whether it generalizes to other stimulus domains. In support of more generic effects, previous studies on auditory processing demonstrated earlier, larger and more robust brainstem responses for musicians relative to non-musicians for both speech and music stimuli (Musacchia et al., 2008; Bidelman and Krishnan, 2010; Bidelman et al., 2011). Moreover, viewing the corresponding videos of the musical instrument in action or facial movements enhanced the temporal and frequency encoding in musicians (Musacchia et al., 2007). Collectively, these results suggest that musical expertise may improve audiovisual processing in a generic fashion at very early processing stages in the brainstem. Based on these results, we may expect that musical expertise fine-tune the temporal integration window generically across multiple stimulus classes such as speech and music.

By contrast, a recent combined psychophysics-fMRI study demonstrated that musicians relative to non-musicians have a significantly narrower temporal integration window for music but not for speech stimuli (Lee and Noppeney, 2011a). Moreover, at the neural level, musicians showed increased audiovisual asynchrony responses and effective connectivity selectively for music but not for speech in a circuitry including the superior temporal sulcus, the premotor cortex and the cerebellum. These results suggest that music practicing may mold audiovisual temporal binding not only via generic mechanisms of perceptual learning but also via more stimulus-specific mechanisms of sensory-motor learning. More specifically, piano music practicing may fine-tune an internal forward model mapping from action plans specific for piano playing onto visible finger movements and sounds. As this internal forward model furnishes more precise estimates of the relative audiovisual timings of music actions, it sensitizes musicians specifically to audiovisual temporal misalignments of music stimuli. Yet, one may argue that natural speech is not an ideal stimulus class to test whether music expertise transfers from music to other stimulus classes, because both musicians and non-musicians are “speech experts” thereby minimizing any additional effects of musical expertise on audiovisual temporal synchrony perception.

To further investigate whether musical expertise shapes temporal binding of non-music stimuli, we presented 21 musicians and 20 non-musicians participants with natural speech, intelligible sinewave analogs of speech, and piano music stimuli at 13 audiovisual stimulus onset asynchronies (±360, ±300 ±240, ±180, ±120, ±60, and 0 ms) (Dixon and Spitz, 1980; Alais and Burr, 2003; Grant et al., 2004; Zampini et al., 2005; Vatakis and Spence, 2006a,b, 2007, 2008a,b; van Wassenhove et al., 2007; Love et al., 2013). On each trial, participants judged the audiovisual synchrony of natural speech, sinewave speech, and piano music stimuli. We have included these three classes of stimuli to elucidate the main factors that determine whether musical expertise generalizes to other classes of stimuli: Natural speech/sinewave speech and piano music are linked to different motor effectors (mouth vs. hand) and thereby rely on different sensori-motor transformations. By contrast, natural speech and intelligible sinewave speech are identical in the visual facial movements and linguistic representations, but differ in their spectrotemporal structure of the auditory input (Remez et al., 1981; Lee and Noppeney, 2011b; Vroomen and Stekelenburg, 2011; Stekelenburg and Vroomen, 2012; Baart et al., 2014). As sinewave speech is generated by replacing the main speech formants with sinewave analogs, sinewave speech obtains a more musical character. Critically, neither musicians nor non-musicians have been exposed to sinewave speech in their natural environment, so that neither of them are sinewave speech experts. Hence, as with other speech transformations such as rotated speech, both groups should have less precise temporal predictions, and hence, yield a wider temporal integration window for sinewave speech than for piano music or natural speech stimuli (see Maier et al., 2011). These aspects render sinewave speech an ideal stimulus to test for transfer effects from music to other stimulus classes.

Finally, previous studies have demonstrated that humans accumulate statistical information over time for deciding whether auditory and visual signals are synchronous or asynchronous (see Vatakis and Spence, 2006a; Maier et al., 2011). We therefore investigated whether the effect of musical expertise on audiovisual synchrony judgments depends on the stimulus duration by presenting participants with short (piano tones, speech syllables) and long stimuli (piano melodies, speech sentences). In our natural environment human observers are predominantly exposed to connected natural speech and piano music (e.g., melodies), thus, musicians should be familiar with the statistical structure of natural speech and piano music stimuli. Therefore, we expected that the effects of musical training would be more pronounced for long duration stimuli (melodies, speech sentences) as compared to short duration stimuli (piano music tones, speech syllables).

## Materials and methods

### Participants

Forty-one German native speakers gave informed consent to participate in the study (mean age ± *SD* = 26 ± 4.9 years). Twenty-one subjects were amateur pianists (mean age ± *SD* = 24.4 ±5.1 years) with an average of 16.1 (*SD* = 5.3) years of experience of piano practicing (mean age of acquisition ± *SD* = 8.2 ± 2.0 years), and they reported that they practiced the piano for an average of 3.48 (*SD* = 1.79) hours per week for the last 3 years. In the non-musicians group, all except three subjects (less than 3 months of music training in drums, bass guitar or flute) had no experience with practicing a musical instrument (mean age ± *SD* = 27.8 ± 4.2 years). The study was approved by the joint human research review committee of the Max Planck Society and the University of Tübingen. A subset of these data (i.e., results for natural speech sentences and melodies) have previously been reported in Lee and Noppeney (2011a, 2014).

### Description of stimuli

Synchronous audiovisual stimuli were recorded from one speaking actress uttering short sentences or one male hand playing on the piano keyboard (showing one octave) using a camcorder (HVX 200 P, Panasonic Corporation, Osaka, Japan; video at 25 frames per second, PAL 768^*^567 pixels) for the visual modality and analog recording for the auditory modality (2 channels, 48 kHz). The speech sentences were short neutral statements in German (4–5 words, 7–9 syllables). The music melodies were generated to match the rhythm and number of syllables to those of the speech sentences. The syllables were *“do,” “re,” “mi,” “fa,” “so,” “la,” “di,” “to,” “bo,” “he,” “zi,” “ka,” “lo,” “ga,” “fi,” “po.”* The piano music tones were *“do,” “re,” “mi,” “fa,” “so,” “la,” “te,” “to.”* Supplementary Material shows the list of speech sentences used in the experiment.

The visual and audio recordings were then digitized into MPEG-4 (H.264) format files. The visual file was first cropped to one single complete visual stimulus (speech or music), preceded and followed by 15 frames of neutral facial expression or a still hand image using Adobe Premier Pro (Adobe Systems, San Jose, CA, USA). We added the additional still images to be able to manipulated audiovisual asynchrony without changing the AV length of the stimuli (please see below and Maier et al., 2011).

To transform the auditory modality of natural speech into sinewave speech, the audio tracks were separated from the video tracks. The auditory natural speech was transformed into sinewave speech by replacing the three formants with sinusoid complexes of three sinusoids that were based on the first three vowel formants (www.lifesci.sussex.ac.uk/home/Chris_Darwin/Praatscripts/SWS). The auditory tracks of sinewave speech were re-combined with the video tracks to create audiovisual movies of sinewave speech. Four sets of stimuli (24 stimuli per set; 8 stimuli per stimulus class) were created; two sets were stimuli of short duration (i.e., syllables or piano tones) and the other two sets were stimuli of long duration (i.e., sentences or melodies). The sets were counter balanced in time across subjects and across groups.

### Experimental design

The experimental paradigm manipulated: (1) stimulus class: audiovisual speech, sinewave analogs of speech with visual utterance movements of natural speech, audiovisual piano music (i.e., piano music with associated hand movements), (2) stimulus duration: short (single syllables and single piano tones; mean duration ± *SD* = 2.38 ± 0.37 s; please note that the duration also include the 15 frame of still images before and after the action sequence), long (sentences and piano melodies; mean duration ± *SD* = 3.56 ± 0.34 s), and (3) audiovisual stimulus onset asynchronies (AV-SOA; ±360, ±300, ±240, ±180, ±120, ±60, 0 ms). Positive values indicated that the visual modality was presented first, whereas negative values indicated that the auditory modality was presented first. More specifically, in synchronous stimuli the temporal relationship between the video and the sound track was kept as obtained from recording and thus reflected the natural audiovisual temporal relationship. In other words, it complied with the natural statistics of audiovisual speech or music. Audiovisual asynchronous stimuli were generated by temporally shifting the onset of the auditory track with respect to the video. Moreover, audiovisual synchrony or asynchrony was then determined by the onset of the facial movements and sound rather than the onset of the video (for similar approach and rationale see Maier et al., 2011).

On each trial, subjects judged whether the audiovisual stimuli were synchronous or asynchronous, in an un-speeded fashion. They completed 8 sessions on 2 separate days. Each stimulus was presented 4 times per session in a randomized manner amounting to 2496 (= 4 sessions for each stimulus duration * 4 times for each stimulus * 8 stimuli per stimulus class * 3 stimulus classes * 13 AV-SOA) trials. The AV-SOA and stimulus class were randomized in each experiment. The stimuli of short and long duration were presented in separate sessions, and the order was counterbalanced across subjects and days. Prior to the experiment, subjects were presented with all stimuli (2 presentations per stimulus), and then tested on their comprehension of the SWS speech sentences by writing down each sentence that they hear.

### Experimental procedure

The AV-SOA of the separate audio and video files was manipulated using Psychophysics Toolbox version 3 (PTB-3) under Matlab 2007b (MathWorks Inc., MA, USA). Visual stimuli (size 8.89° * 7° visual angle) were projected using a CRT monitor (Sony Trinitron, Tokyo, Japan) at refresh rate of 100 Hz, and subjects' heads were stabilized using a chin rest. Auditory stimuli were presented at ~75 dB SPL via headphones.

### Data analysis

For each subject and condition, the proportion of synchronous responses (PSR) was computed for each of the 13 AV-SOA levels. To refrain from making any distributional assumptions, the psychometric function was estimated using a non-parametric approach based on local linear fitting methods (Zychaluk and Foster, 2009). The bandwidth for the local quadratic fitting was optimized individually for each subject in a cross-validation procedure. We characterized the psychometric functions by the width of the temporal integration window, as determined by the integral of the psychometric function between −360 and +360 ms (after subtracting the difference between one and the maximum from all values of the fitted psychometric function, so that the maximum of all functions was set to one).

To evaluate whether there are any differences in the widths of the temporal integration window between groups, stimulus duration and stimulus class, mixed design ANOVAs were performed with stimulus duration (short, long) and stimulus class (natural speech, sinewave speech, piano music) as within-subject factors, and group (non-musicians, musicians) as a between-subject factor. The results of the ANOVAs are reported after Greenhouse-Geisser correction (when applicable).

## Results

After presenting subjects with all stimuli twice (before the main study), we tested them on the comprehension of sinewave speech sentences and syllables. Participants obtained 100% accuracy before the start of the experiment. This ensured that the intelligibility of sinewave speech stimuli could be considered speech-like for the main experiment.

**Figure 1:**
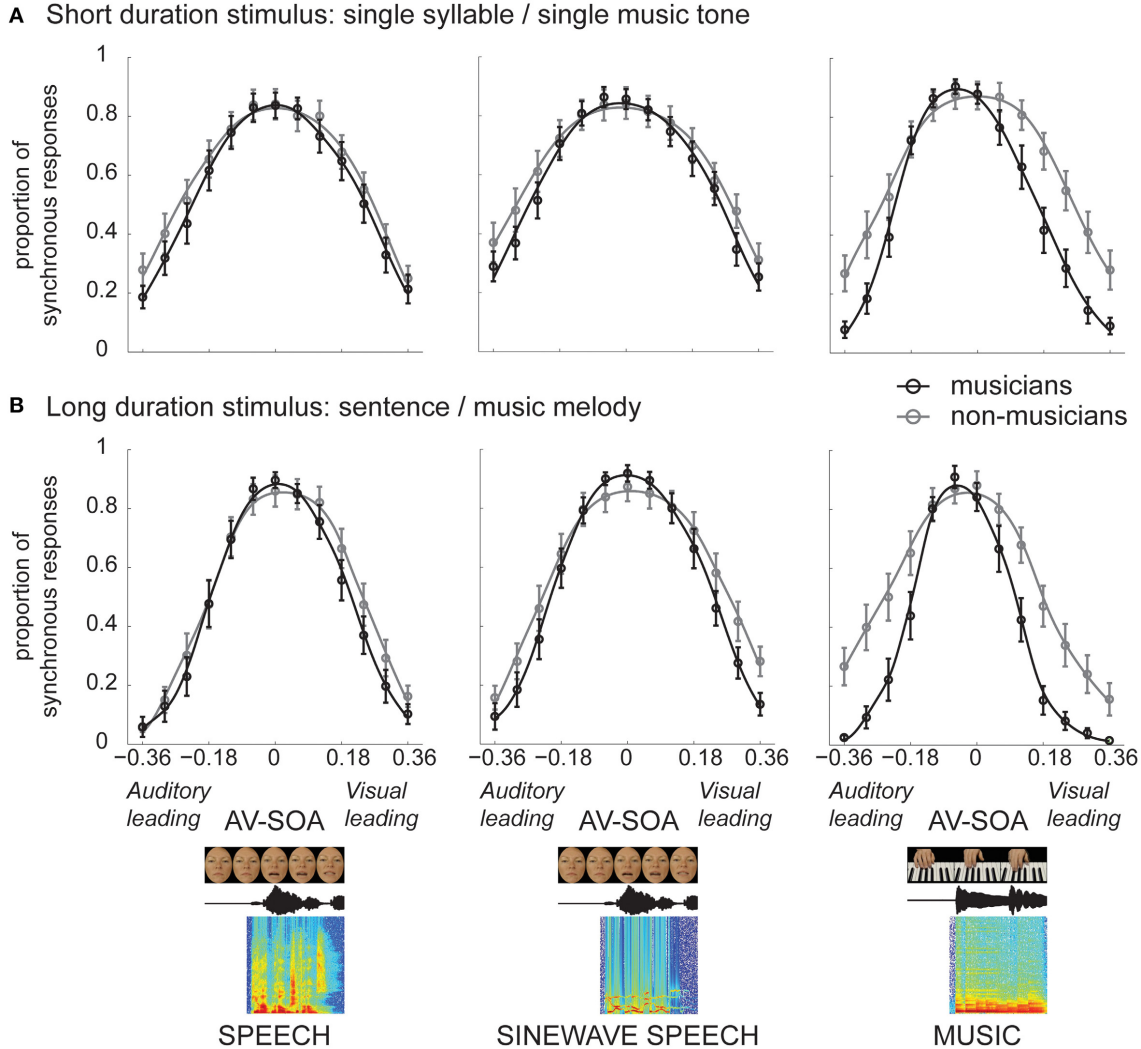
**The psychometric functions for speech, sinewave speech, and piano music in non-musicians and musicians for (A) short duration stimulus (syllables or single music tones), and (B) long duration stimulus (sentences or melodies)**.

**Figure 2:**
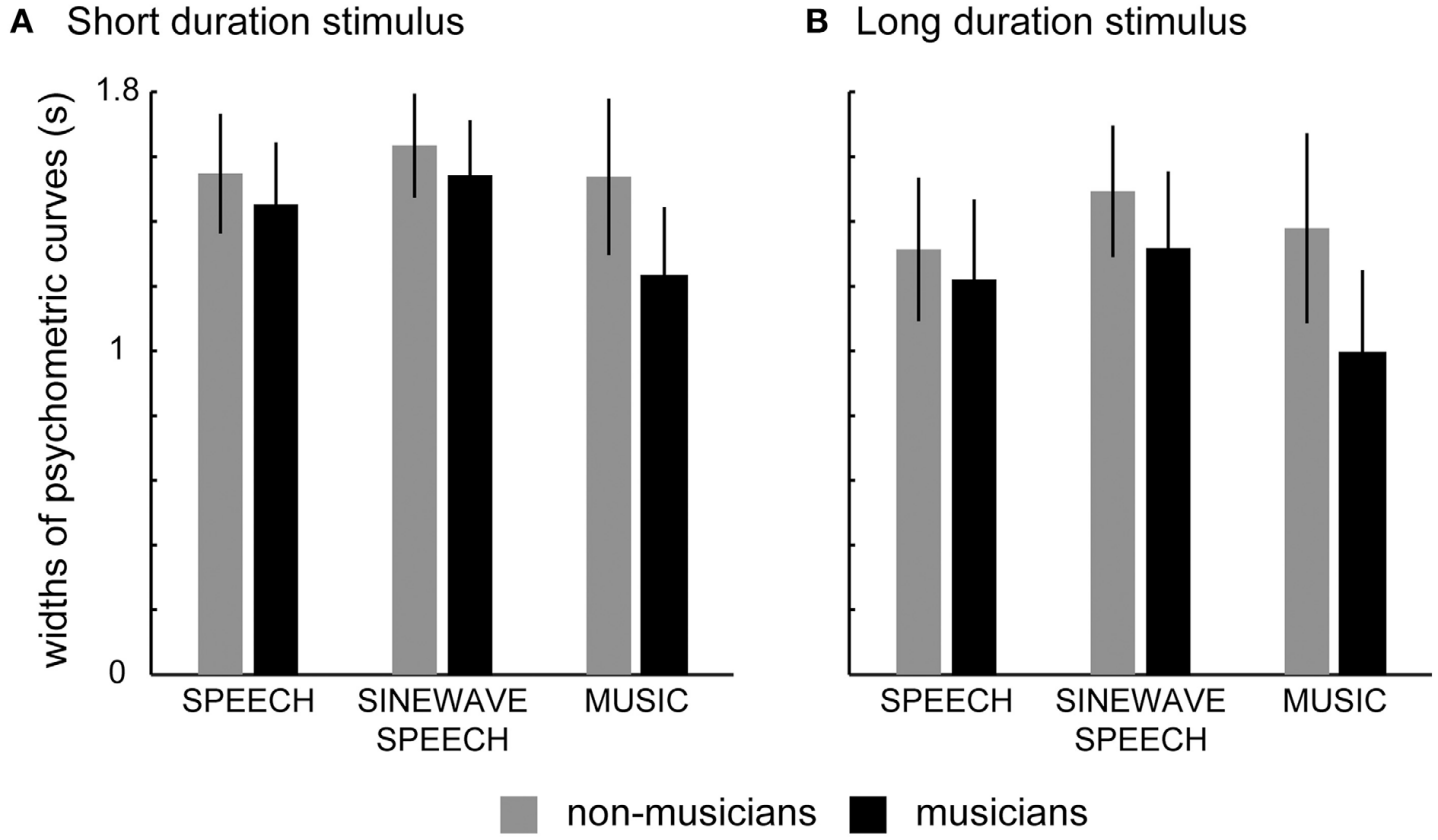
**Bar plots showing mean (across subjects' mean) of the widths of the temporal integration windows for speech, sinewave speech, and piano music in non-musicians and musicians for (A) short duration stimulus (syllables or single music tones), and (B) long duration stimulus (sentences or melodies)**. Error bars represent 1 *SD* (standard deviation).

Subjects' PSR for each condition was computed, and psychometric functions were estimated using a non-parametric local quadratic fitting method (Zychaluk and Foster, 2009). Figure 1 shows the psychometric functions (averaged across subjects) separately for each condition in the musician and non-musician groups. Figure 2 shows the bar plots of the mean (across subjects' mean) widths of the temporal integration windows for each condition in the musician and non-musician groups. The 2 (group: non-musicians, musicians) × 2 (stimulus duration: short, long) × 3 (natural speech, sinewave speech, piano music) mixed design ANOVA on the widths of the temporal integration windows (Table 1) revealed a main effect of stimulus duration. Thus, as previously suggested, participants accumulate information over time and thereby obtain more precise temporal estimates for long (i.e., melodies or sentences) relative to short duration stimuli (i.e., piano music tones or syllables) (Maier et al., 2011). Another previous study has reported the opposite finding, i.e., smaller temporal integration windows for syllables as compared to sentences (Vatakis and Spence, 2006a). Vatakis and Spence (2006a) have attributed their results to increased low-level spatiotemporal correlations or increased likelihood of binding attributable to the assumption of “unity” for long relative to short stimuli. However, this previous study differs from the current study in many aspects: (i) they used a temporal order judgment task, (ii) they included only very few stimuli (e.g., only two particular sentences), which makes generalization and interpretation difficult, and (iii) they investigated syllables and sentences in distinct sets of subjects and did not report a formal statistical comparison. For further discussion regarding the issue of stimulus duration, please refer to our previous study that aimed to address the influence of stimulus duration on audiovisual temporal integration window (Maier et al., 2011).

**Table 1:** **Results of the mixed ANOVA on the widths of temporal integration windows with stimulus duration (short, long) and stimulus class (speech, sinewave speech, music) as within-subject factors, and group (non-musicians, musicians) as between-subject factor**.

**Main effects of:**		
Group	*****F***_**(1, 39)**_ = 10.08**	*****p*** = **0.003****
Stimulus duration	*****F***_**(1, 39)**_ = 129.5**	*****p*** < **0.001****
Stimulus class	*****F***_**(1.48, 57.8)**_ = 53.5**	*****p*** < **0.001****
**Interactions of:**		
Group * stimulus duration	*F*_(1, 39)_ = 2.20	*p* = 0.146
Group * stimulus class	*****F***_**(1.48, 57.8)**_ = 22.0**	*****p*** < **0.001****
Stimulus duration * stimulus class	*F*_(1.46, 56.9)_ = 1.60	*p* = 0.215
Group * stimulus duration * stimulus class	*F*_(1.46, 56.9)_ = 1.44	*p* = 0.243

*Significant effects are indicated in bold*.

**Figure 3:**
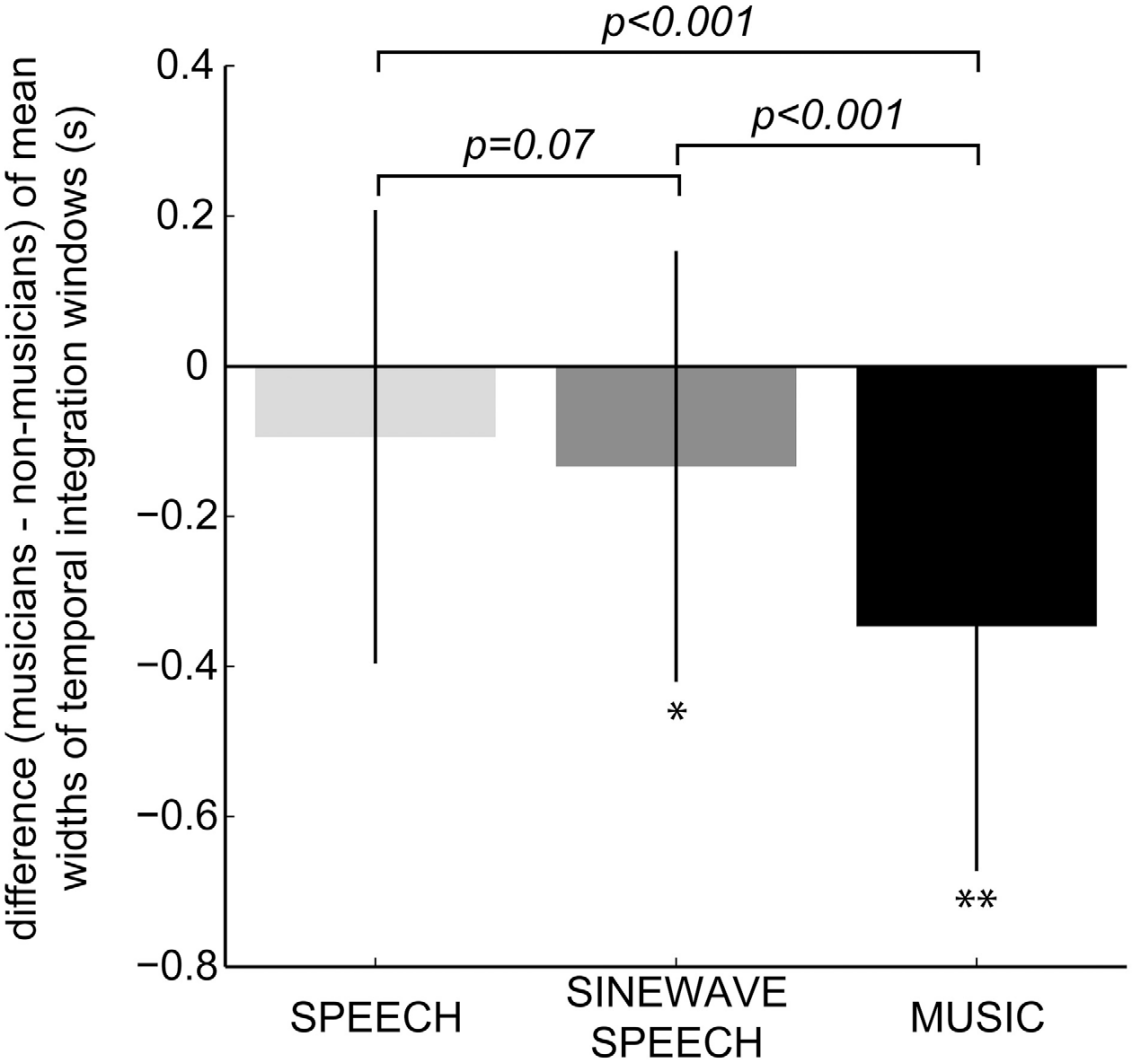
**Bar plots showing the musical expertise effect, i.e., difference of the mean widths of the temporal integration windows for musicians vs. non-musicians (musicians – non-musicians; a negative value indicated that musicians relative to non-musicians exhibited a narrower temporal integration window)**. Error bars represent 1 *SD* (standard deviation). Significance was calculated using one-tailed two samples *t*-tests on the difference of the mean widths of the temporal integration windows for musicians vs. non-musicians (^*^p < 0.05; ^**^p < 0.001). Additionally, *p*-values (one-tailed) of one-tailed two samples *t*-tests of the musical expertise effects for sinewave speech > natural speech, piano music > sinewave speech, and piano music > natural speech are shown.

Critically, we also observed main effects of stimulus class and group, as well as an interaction between stimulus class and group. As expected, music practice influenced musicians' temporal integration window in a stimulus-dependent fashion and had the strongest effect on piano music stimuli. Thus, as shown in Figure 3, the difference in widths of the temporal integration windows for musicians and non-musicians (i.e., the musical expertise effect) was the largest for piano music stimuli. Contrary to our initial hypothesis, we did not observe a significant three-way interaction of stimulus duration, stimulus class and group, a two-way interaction between group and duration, or a two-way interaction between stimulus class and duration. Therefore, we pooled the widths of the temporal integration windows across stimulus duration for natural speech, sinewave speech and piano music, and examined the effect of musical expertise for each stimulus class by computing the difference of the mean widths of the temporal integration windows for musicians relative to non-musicians (i.e., musicians – non-musicians). Figure 3 depicts the bar plots for the difference (musicians – non-musicians) of the mean widths of temporal integration windows for natural speech, sinewave speech and piano music. Specifically, we tested whether the musical expertise effect (i.e., the difference for musicians – non-musicians) on the widths of temporal integration windows was significantly greater than zero. *Post-hoc* two samples *t*-tests (one-tailed) for each stimulus class revealed that musicians relative to non-musicians exhibited significantly narrower temporal integration windows for sinewave speech [*t*_(39)_ = 2.34, *p* = 0.025; *one-tailed p* = 0.01] and piano music [*t*_(39)_ = 4.74, *p* < 0.001], and a marginal significance for natural speech [*t*_(39)_ = 1.49, *p* = 0.14; *one-tailed p* = 0.07]. Further, as illustrated in Figure 3, we observed a gradient of musical expertise effects for piano music > sinewave speech > natural speech. This observation was confirmed statistically by *post-hoc* testing for the three interactions that selectively compare the musical expertise effect across two stimulus classes (e.g., musicians – non-musicians for piano music – natural speech). These tests demonstrated that musicians relative to non-musicians exhibited narrower temporal integration windows for piano music > natural speech [*t*_(39)_ = 5.41, *p* < 0.001] and music > sinewave speech [*t*_(39)_ = 4.58, *p* < 0.001], and a marginal significance for sinewave speech > natural speech [*t*_(39)_ = 1.51, *p* = 0.14; *one-tailed p* = 0.07]. A one-tailed *t*-test can be adopted, because we would expect a stronger musical expertise effect for sinewave speech than natural speech stimuli (see Introduction).

Collectively, these results demonstrate that the effect of musical expertise was most pronounced for piano music stimuli, and it also generalized to sinewave speech and to a marginally significant extent to natural speech stimuli. However, contrary to our expectations, the musical expertise effect did not depend on stimulus duration. This suggests that even short stimuli provided sufficient statistical structure that enabled musicians to generate more precise estimates of the relative timing of the audiovisual signals.

### Correlation analyses of the widths of audiovisual temporal integration windows with age of acquisition and amount of practice

The narrowing of the temporal integration window for musicians may result from innately specified (e.g., genetic) differences between musicians and non-musicians. Alternatively, it may reflect plasticity induced by long-term musical training (Munte, 2002; Zatorre et al., 2007). In the latter case, the narrowing of the temporal integration may depend on the amount of time that musicians spent on piano practicing. Further, the effect of music practice may also interact with neurodevelopment and be most pronounced when children start practicing a musical instrument early in life. In this case, the effect of music practicing should depend on the age at which musicians started piano practicing. Effects of age of acquisition would for instance be observed if piano practicing relies on mechanisms that need to be fine-tuned during sensitive periods in neurodevelopment.

**Figure 4:**
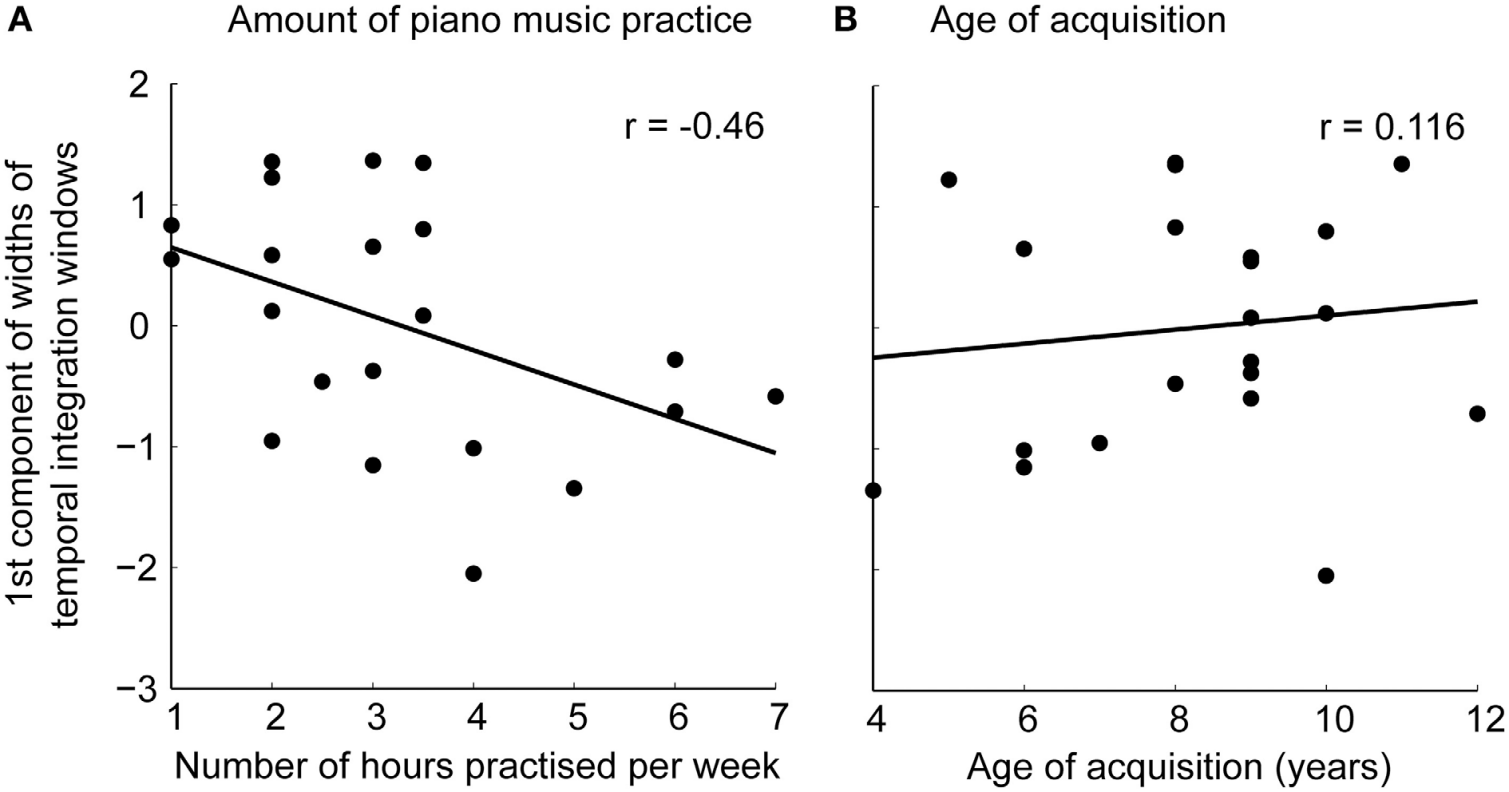
**Scatterplots showing correlations of the first component of the widths of temporal integration windows across all stimulus classes with (A) amount of piano music practice over the past 3 years and (B) age of acquisition**.

To test whether the narrowing of temporal integration window results from training-induced plasticity, we performed separate correlation analyses testing for a correlation between the width of the psychometric function with (i) age of acquisition or (ii) amount of weekly music practice (in hours) during the past 3 years as predictors. As the widths of temporal integration windows were highly correlated across the different conditions over subjects, we first performed a principal component analysis on the subject-specific widths across all conditions for data reduction. The first component explained 76.3% of the total variance of all the widths, while the second component explained 9.1% and the third component explained 6.2% of the total variance of all the widths. Thus, as the 2nd component explained only a negligible amount of variance in the data, we extracted and correlated only the first component with age of acquisition and amount of weekly piano music practice during the past 3 years. A significant correlation was found for the first component and amount of weekly piano music practice during the past 3 years [*r*_(21)_ = −0.46, *p* = 0.037] (Figure 4A), whereas no significant correlation was found for the first component and age of acquisition [*r*_(21)_ = 0.116, *p* = 0.617] (Figure 4B). Specifically, the more the musicians practiced piano, the narrower their temporal integration windows were (i.e., the more sensitive they became to the temporal misalignment of auditory and visual signals).

## Discussion

Our results demonstrate that long-term music training shapes the temporal integration window in a stimulus-dependent fashion. Musicians, relative to non-musicians, exhibited a narrower temporal integration window predominantly for piano music and to some extent also for sinewave speech with a marginally significant trend for natural speech. Moreover, the amount of weekly piano music practice in the past 3 years correlated with the widths of the temporal integration windows across all stimulus classes. In other words, the more musicians practiced piano in the past 3 years, the more sensitive they became to audiovisual temporal misalignments for natural speech, sinewave speech, and music. Collectively, our results demonstrate that music practice furnishes more precise estimates regarding the relative timings of the audiovisual signals predominantly for music, yet this effect also transferred partly to speech.

Accumulating evidence suggests that music practicing and perceptual learning can influence how human observers temporally bind signals from multiple senses. For instance, a recent psychophysics study demonstrated that musical expertise narrows the temporal integration window for music (Petrini et al., 2009). Yet, this study included only music stimuli. Thus, an unresolved question is to what extent these music or perceptual learning effects are specific to the particular stimulus class trained or whether they can generalize to other stimulus classes. In support of generic mechanisms of musical expertise, electrophysiological recording demonstrated earlier and larger brain stem responses for musicians relative to non-musicians for both speech and music (Musacchia et al., 2007, 2008; Bidelman and Krishnan, 2010; Bidelman et al., 2011). By contrast, a recent neuroimaging study demonstrated that music practice fine-tunes the temporal integration window predominantly for piano music via engagement of a premotor-cerebellar circuitry (Lee and Noppeney, 2011a).

The current study therefore revisited the question of whether music practice influences audiovisual temporal integration not only of the trained piano music stimuli but also untrained stimulus classes. To this aim, we included natural speech and intelligible sinewave speech signals where the main speech formants have been replaced by sinewave analogs, thereby giving sinewave speech a musical character. Critically, even though the sinewave speech transformation preserved stimulus intelligibility, it introduced a novel mapping between auditory and visual signals. Indeed, as expected, this novel audiovisual mapping made it harder for participants to discriminate between synchronous and asynchronous audiovisual sinewave speech as indicated by a broader integration window for sinewave speech as compared to natural speech (for related findings on rotated speech, see Maier et al., 2011). Thus, the comparison of piano music, sinewave speech and natural speech stimuli enabled us to better characterize to which extent music practice effects transfer to other stimulus classes.

Our results replicate that music expertise shapes temporal binding of audiovisual signals in a stimulus-dependent fashion as indicated by a significant interaction between stimulus class and group. Thus, we observed a gradient of musical expertise effects decreasing from piano music > sinewave speech > natural speech. Nevertheless, the effects of musical expertise on the temporal integration window of other stimulus classes such as sinewave speech or natural speech were still significant. The gradient of musical expertise effect across stimulus classes may be accounted for by two different explanatory frameworks:

First, audiovisual temporal perception may be mediated by only one domain-general mechanism that is engaged by all stimulus classes. Since this domain-general system can be fine-tuned via training to the statistics of a particular stimulus class, the musical expertise effect varies across stimulus classes in a gradual fashion. Thus, pianists would be particularly sensitive to audiovisual asynchronies of piano music stimuli, because the domain-general system has been fine-tuned to the audiovisual temporal statistics of piano music. Yet, transfer effects of musical expertise also emerge, because other stimulus classes can benefit from the fine-tuning of a domain-general system.

Alternatively, the gradient in musical expertise effects may be explained by the concurrent engagement of domain-general and stimulus-specific mechanisms. Domain-general mechanisms have been proposed by a vast number of studies showing musical expertise effects that generalize across music and speech stimuli at the behavioral (Chandrasekaran et al., 2009; Elmer et al., 2012, 2013; Marie et al., 2012; Asaridou and Mcqueen, 2013) or neural level (Musacchia et al., 2008; Bidelman and Krishnan, 2010; Bidelman et al., 2011; Elmer et al., 2012, 2013; Marie et al., 2012). Conversely, we recently showed that music practice sharpens the temporal integration window predominantly for music via premotor-cerebellar circuitry (Lee and Noppeney, 2011a) and proposed that piano practicing may mold the audiovisual temporal integration by training an internal forward model that maps from motor actions (e.g., piano practicing) to its sensory consequences in vision (i.e., finger movements) and audition (e.g., piano sound when hitting the key). Thus, a combination of such a domain-general and a stimulus-dependent sensory-motor mechanism may better explain the transfer of musical expertise effects to other stimulus classes such as sinewave speech in a gradual fashion.

The comparison of musicians and non-musicians cannot resolve ambiguities about whether or not the mechanisms are innately specified or truly reflect experience-dependent plasticity. For instance, amateur musicians may have chosen to practice a musical instrument, because they were inherently better at temporal perception via innate mechanisms. Yet, if musical expertise depends on experience-dependent mechanisms, we would expect that the temporal integration window decrease with the amount of practice. Moreover, if these experience-dependent mechanisms interact with development (e.g., sensitive periods), the integration window should also be influenced by the age at which participants started practicing a musical instrument. Our results demonstrate that indeed the amount of weekly piano practicing in the past 3 years correlates negatively with the musicians' widths of the temporal integration windows—more specifically the first principal component over widths across all conditions. In other words, the more musicians practiced piano, the more sensitive they were to audiovisual temporal misalignments of speech and piano music stimuli. Surprisingly, the age at which musicians started piano practicing did not correlate significantly with the widths of their temporal integration windows. This dissociation suggests that piano practicing shapes audiovisual temporal integration and sensitivity to temporal misalignments via experience-dependent mechanisms that either do not critically interact with neurodevelopment or are bound to sensitive periods in very early development (i.e., before the age of four when the first of our participants started piano practicing). Yet, our results are based on correlative methods. To further substantiate our conclusions, prospective longitudinal studies are required that investigate the change in the temporal integration window as a function of piano music practicing [e.g., 2 (piano practicing vs. other activity) × 2 (before, after training) factorial design].

In conclusion, our results suggest that piano music practicing shapes the temporal integration of audiovisual signals via experience-dependent plasticity. While musical expertise strongly narrows the width of the temporal integration window for piano music, the effect transfers to non-music stimuli such as sinewave speech and a non-significant trend to natural speech. Thus, piano music practicing affects temporal binding either via mechanisms that are specialized predominantly for music but transfer at least in part to other stimulus classes. Alternatively, piano music practicing influences temporal binding of audiovisual signals via multiple mechanisms including stimulus-specific (i.e., specialized for music stimuli) and generic mechanisms (e.g., perceptual learning).

### Conflict of interest statement

The authors declare that the research was conducted in the absence of any commercial or financial relationships that could be construed as a potential conflict of interest.
