# Supplementary material for: Music expertise shapes audiovisual temporal integration windows for speech, sinewave speech, and music
Source: Front Psychol. 2014 Aug 7;5:868. doi: 10.3389/fpsyg.2014.00868 (PMC4124486; doi:10.3389/fpsyg.2014.00868)
Supplement: Supplementary file 1 [file Presentation1.PDF]

Appendix A. List of speech and sinewave sentences.

1. Piraten rauben Schiffe aus.
2. Pollen fliegen durch die Luft.
3. Taeter meiden ihre Opfer.
4. Tische haben vier Beine.
5. Der Postbote bringt die Post.
6. Die Ampel regelt den Verkehr.
7. Katzen haben neun Leben.
8. Kugeln haben eine runde Form.
9. Gaense haben weisse Federn.
10. Geister machen vielen Menschen Angst.
11. Inder haben dunkle Haut.
12. Otter haben weiches Fell.
13. Baeren haben grosse Tatzen.
14. Blumen haben bunte Blueten.
15. Nachspeisen enthalten oft viel Fett.
16. Nonnen tragen schwarze Kutten.
